# The legacy effects of electromagnetic fields on bone marrow mesenchymal stem cell self-renewal and multiple differentiation potential

**DOI:** 10.1186/s13287-018-0955-5

**Published:** 2018-08-09

**Authors:** Chang Tu, Yifan Xiao, Yongzhuang Ma, Hua Wu, Mingyu Song

**Affiliations:** 1 Department of Orthopedics, Tongji Hospital, Tongji Medical College, Huazhong University of Science and Technology, Wuhan, Hubei People’s Republic of China; 2 Department of Immunology, School of Basic Medicine, Tongji Medical College, Huazhong University of Science and Technology, Wuhan, Hubei People’s Republic of China; 3 Department of Obstetrics and Gynecology, Tongji Hospital, Tongji Medical College, Huazhong University of Science and Technology, Wuhan, Hubei People’s Republic of China

**Keywords:** Electromagnetic fields, Legacy effects, Mesenchymal stem cells, Proliferation, Differentiation, Cell sheets

## Abstract

**Background:**

The effects of electromagnetic fields (EMF) on bone nonunion have been reported for many years. Many studies and randomized controlled trials have demonstrated that EMF exhibited benefits in curing delayed union and nonunion of long bone fractures. Most of them focused on the immediate effects, while the legacy effects of EMF remain poorly investigated.

**Methods:**

In this study, rat bone marrow mesenchymal stem cells (BMSCs) were treated with EMF, and after a period of time the BMSC proliferation and differentiation were detected. Additionally, BMSC sheets with or without EMF treatment were transplanted into the rat tibia fracture nonunion models. The bone formation was evaluated after 2, 4, and 6 weeks.

**Results:**

Our results showed that the proliferation capacity of BMSCs was heightened after EMF pretreatment. Over a period of time of EMF pretreatment, the capacities of osteogenic and chondrogenic differentiation were enhanced, while adipogenic differentiation was weakened. BMSC sheets pretreated with EMF could better promote the healing of tibia fracture in rats, compared to BMSC sheets alone. Furthermore, significantly higher values of radiographic grading scores were observed in the EMF group.

**Conclusions:**

EMF has lasting effects on the proliferation and differentiation of BMSCs, and together with cell sheet technology can provide a new method for the treatment of fracture nonunion.

## Background

Bone nonunion is a clinical challenge with an incidence of 5–10% [1]. Electromagnetic fields (EMF) have been studied for bone disorders for many years [2, 3]. Several research studies have shown that EMF with different parameters had various effects on bone problems [4–6]. However, the current study mainly focuses on the immediate effect of EMF, namely detection and comparison immediately after the end of the stimulation with EMF; a continuous impact of EMF on bone-related incidences has been reported rarely. It has been reported that patients with osteoporosis still receive therapeutic benefits after the end of treatment with EMF [7, 8]. Moreover, EMF exposure was revealed to induce epigenetic changes on somatic cells, providing an efficient tool for epigenetic reprogramming [9]. Prolonged exposure to EMF induces persistent changes in neuronal activity [10]. Therefore, it is high time for us to explore the legacy effects of EMF treatment.

Tissue-engineered bone combined with stem cells has great potential in the treatment of bone nonunion [11]. Due to the unsatisfactory biocompatibility of graft materials [12, 13], it is of vital importance to find an alternative approach. The cell sheet is a technique based on culturing cells in hyperconfluency until they form an intact cell sheet [14]. This technique avoids enzymatic digestion of cells, preserving intercellular connections and extracellular matrix [15]. A fabricated single cell sheet has been applied into skin, cornea, periodontal ligament, or mucosa reconstruction [15, 16]. In a rat bone nonunion model, a BMSC sheet as a scaffold-free graft can obviously promote fracture healing [17, 18]. However, due to the limitations of the cell sheet source and the uncontrollable cell differentiation, various studies seek to explore regulatory approaches including vitamin C treatment, electropathy, a pH change-induced method, and magnetic treatment [19, 20].

Bone marrow mesenchymal stem cells (BMSCs) are multipotent progenitors with self-replication and multilineage differentiation capacity [21]. In bone tissue engineering or bone-related disease models, BMSCs are often used for research. To value the effects of EMF in vitro, lots of studies combined EMF treatment with BMSCs, and the proliferation and differentiation capacity were explored [22]. Because of their limited source, BMSCs should be expanded in vitro before application. However, during long-term in-vitro culture, the cell morphology changes and the expression of BMSC specific surface antigen is reduced [23]. Moreover, the self-replication and multidirectional differentiation capacity of BMSCs decreases over time [24]. This will lead to a reduction in the therapeutic effect of BMSCs and hinder their clinical application. Many stimuli including EMF had been unearthed to solve this problem, and if EMF exposure can have a lasting effect on the proliferation and differentiation of BMSCs, the current problem will be partially answered.

In this study, sinusoidal EMF (SEMF) (15 Hz, 1 mT, 4 h/day) were selected as a stimulus. The legacy effects of EMF on BMSC proliferation and differentiation were explored in vitro. In vivo, BMSC sheets with or without pretreatment with EMF were used to be implanted into the defects and the bone formation was evaluated. We expected to explore a new method for bone nonunion treatment.

## Methods

### Reagents

TRIzol reagent was procured from Invitrogen (Carlsbad, CA, USA). Dulbecco’s modified Eagle’s medium F12 (DMEM/F12) was purchased from HyClone (Grand Island, NY, USA). MSC osteogenic differential medium, adipogenic differential medium, and chondrogenic differential medium were obtained from Cyagen Biosciences Inc. (USA). Antibodies against OPN, SOX9, AIPOQ, and PPARγ2 were purchased from Abcam (Cambridge, UK). Antibody specific for Col2 was obtained from Cell Signaling Technology (Beverly, MA, USA). Antibodies against GAPDH, RUNX2, secondary antibodies, and cell counting kit-8 (CCK-8) were provided by Boster (Wuhan, China).

### BMSC culture and BMSC sheet preparation

Sprague-Dawley rats 6–8 weeks old (male, 60–100 g) were obtained from the Laboratory Animal Center of Tongji Hospital of Hubei province in China. All experimental procedures followed the Guidelines of Animal Care and Use Committee for Teaching and Research of Huazhong University of Science and Technology. Rat BMSCs were isolated according to the process described previously [25]. Briefly, BMSCs were collected by flushing the bone marrow outside the femurs and tibias of rats with an 18-gauge sterile needle. The bone marrow was suspended in growth medium (GM) consisting of DMEM/F12 medium supplemented with 10% fetal bovine serum (FBS; Gibco, NY, USA), 100 U/ml penicillin, and 100 U/ml streptomycin (Sigma-Aldrich, St. Louis, MO, USA). The isolated cells were then washed twice with phosphate-buffered saline (PBS, pH 7.4), resuspended in GM, plated at a density of 1 × 10^6^ cells/cm^2^ in 25-cm^2^ flasks, and cultured at 37 °C in 5% CO_2_. After every passage, nonadherent cells were removed. The second or third passage was used for subsequent experiments.

For osteogenic differentiation, adipogenic differentiation, and chondrogenic differentiation, BMSCs were cultured with inductive medium respectively according to the protocol from Cyagen Biosciences.

For BMSC sheet harvest, third-passage cells were seeded at 1 × 10^4^ cells/cm^2^ onto 10-cm dishes. Cells were cultured with GM and the GM was refreshed every 3 days. After approximately 14 days, the cells reached hyperconfluence and were lifted as a cell sheet using a scraper.

### EMF exposure

**Fig. 1:**
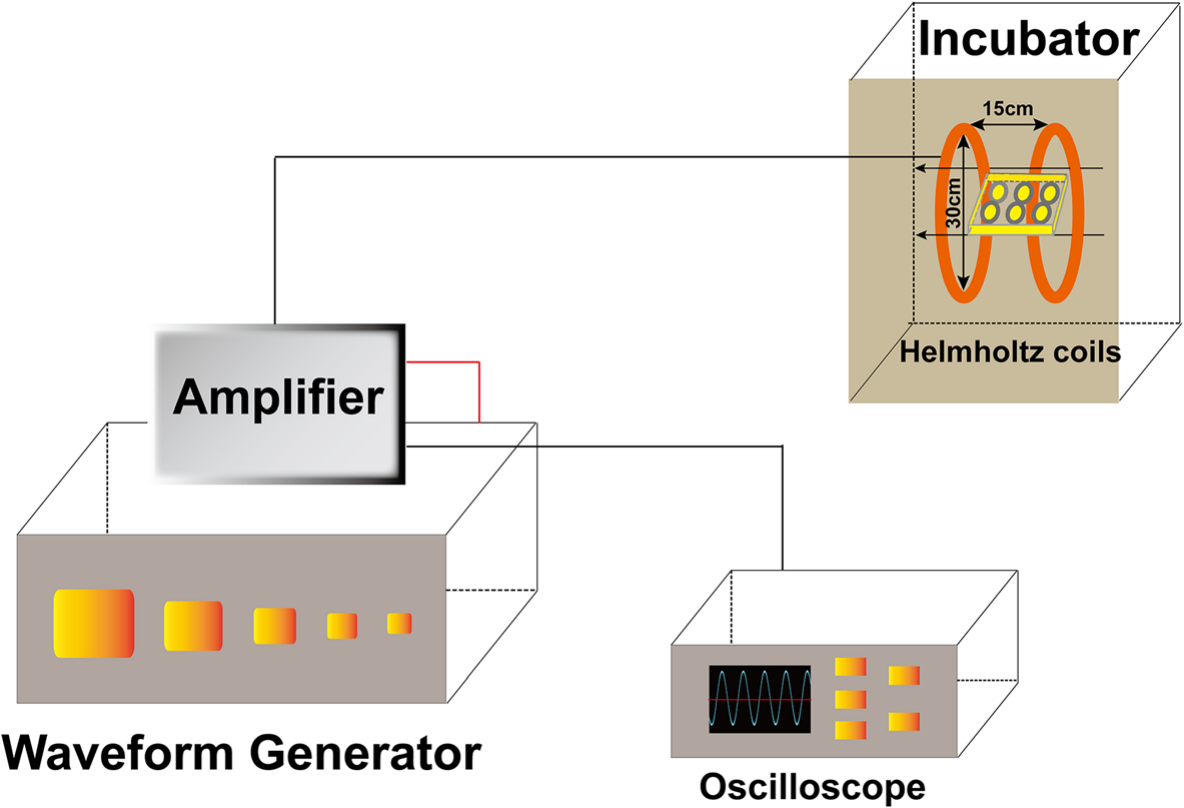
Schematic diagram of EMF device. The facility consists of four main parts: waveform generator, amplifier, two Helmholtz coils, and oscilloscope. EMF frequency verified by oscilloscope as consistent and sinusoidal. Helmholtz coils which produced uniform EMF placed in an incubator (37 °C, 5% CO_2_), and cell culture plates placed in center of coils

The EMF facility was designed and manufactured by Naval Engineering University of China (Fig. 1). Briefly, the device was composed of a waveform generator, an amplifier, an oscilloscope, and Helmholtz coils. Signals were emitted by the waveform generator. With the help of an amplifier and oscilloscope, the signals were transferred to the coils. The coils producing EMF were placed in a 5% CO_2_ incubator. In our study, we used sinusoidal EMF (SEMF) and the parameters were 1 mT, 15 Hz, 4 h/day. The temperatures were measured daily inside the incubators with or without the EMF device with a hydro-thermometer (AR827; Smart Sensor, Hong Kong, China), and the differences were within 0.2–0.8 °C. During exposure, BMSCs cultured in flasks or plates were placed in the center of the coils. Control samples were kept in the same conditions without exposure to SEMF.

### Cell proliferation assay

For the EMF group, rat BMSCs of passage 2 were treated with SEMF (1 mT, 15 Hz, 4 h/day) for 7 days. After EMF exposure, cells were seeded in 96-well plates at a density of 1.5 × 10^3^ cells/well. Cell proliferation was analyzed with a cell counting kit-8 (CCK-8; Boster) according to the standard protocol. Briefly, 10 μl CCK-8 solution with 100 μl GM was added into each well. After incubation for 2 h, the optical density (OD) value was read by a microplate reader (Bio-TEK Instruments, Winooski, VT, USA) at 450 nm. The assay was performed from day 0 to day 6. For the Control group, BMSCs were cultured for the same duration as the EMF group without EMF exposure.

### Fibroblastic colony-forming assay

For the EMF group, rat BMSCs of passage 2 were treated by SEMF (1 mT, 15 Hz, 4 h/day) for 7 days. After EMF exposure, 4 × 10^2^ cells were seeded onto a 3.5-cm dish containing GM. The medium was half exchanged every 3 days. After culturing for 7 days, the dishes were washed with PBS and the BMSCs were fixed with 4% paraformaldehyde (Sigma-Aldrich). Then, the cells were stained by 0.1% toluidine blue solution (Sigma-Aldrich). Colonies containing more than 50 cells were counted and the colony-forming ratio was calculated as colony number/400. For the Control group, cells were controlled to the same passage and same culturing duration without EMF treatment.

### Alizarin Red S staining

For the EMF group, BMSCs of passage 3 were seeded in 3.5-cm plastic dishes with GM. The cells were treated with SEMF (1 mT, 15 Hz, 4 h/day) for 7 days. After EMF treatment, the GM was replaced by osteogenic differential medium. Then the BMSCs were cultured for another 14 days. The inductive medium was refreshed following the protocol provided by Cyagen Biosciences. When the culturing was finished, cells were rinsed with PBS, fixed with 4% paraformaldehyde, and washed with deionized water. We stained the cells with 40 mM Alizarin Red S (Sigma-Aldrich) and photographed the cell staining. For the Control group, BMSCs were kept in the same conditions except for the EMF exposure.

### Alcian Blue staining

For the EMF groups, BMSCs of passage 3 were seeded in 3.5-cm plastic dishes with GM. The cells were treated with SEMF (1 mT, 15 Hz, 4 h/day) for 7 days. The GM was replaced by chondrogenic differential medium after the EMF exposure. Then the cells were cultured for another 14 days. The inductive medium was refreshed according to the process from Cyagen Biosciences. When finishing culturing, cells were washed with PBS, fixed with 4% paraformaldehyde, and rinsed with distilled water. Induced cells were stained with Alcian Blue solution (pH 2.5; Sigma-Aldrich) and the cell staining was photographed. For the Control groups, BMSCs were controlled to the same conditions without EMF treatment.

### Oil Red O staining

For the EMF group, third-passage BMSCs were seeded in 3.5-cm plastic dishes with GM. After treatment of SEMF (1 mT, 15 Hz, 4 h/day) for 7 days, the GM was replaced by adipogenic differential medium. We cultured the cells for another 14 days, and the inductive medium was refreshed following the process from Cyagen Biosciences. When the culturing was finished, cells were rinsed with PBS, fixed with 4% paraformaldehyde, and washed with distilled water and 60% isopropanol. Induced cells were stained with filtered Oil Red O (Sigma-Aldrich) at a ratio of 60% Oil Red O stock solution to 40% distilled water. After staining, we took the photographs with an inverted microscope (Nikon). For the Control group, culturing conditions were kept the same except for the EMF exposure.

### Quantitative real-time PCR

Total RNA was extracted by TRIzol reagent following the manufacturer’s instructions. RNA sample purity and concentration were determined spectroscopically. Then 3μg RNA was reverse-transcribed to cDNA with an EasyScript First-Strand cDNA Synthesis SuperMix kit (TransGen Biotech, Beijing, China) and used for RT-PCR. The expression of mRNA was evaluated by a Bio-Rad myiQ2 thermal cycler (Bio-Rad, Hercules, CA, USA). GAPDH was used as the internal control for target mRNA. RT-qPCR primers used in this study are presented in Table 1. All primers were synthesized by Invitrogen. qPCR cycling conditions was 95 °C for 30 s followed by 40 cycles of 94 °C for 5 s and 60 °C for 35 s. The 2^–△△Ct^ method was used to analyze the relative expression of targeted mRNA expression. Compared to the Control group, the EMF group was pretreated with EMF for 7 days before culturing in inductive medium.

**Table 1:** Specific primers used in this study

Gene	Primer sequences
*GAPDH*	Forward: 5′-AACGACCCCTTCATTGACCTC-3′
	Reverse: 5′-CCTTGACTGTGCCGTTGAACT-3′
*RUNX2*	Forward: 5′-CTACTCTGCCGAGCTACGAAAT-3′
	Reverse: 5′-TCTGTCTGTGCCTTCTTGGTTC-3′
*OPN*	Forward: 5′-CAAGGACCAACTACAACCA-3′
	Reverse: 5′-GGAGACAGGAGGCAAGG-3′
*ALP*	Forward: 5′-CAAGGACCAACTACAACCA-3′
	Reverse: 5′-AGGGAAGGGTCAGTCAGGTT-3′
*OCN*	Forward: 5′-GGAGGGCAGTAAGGTGGTGA-3′
	Reverse: 5′-GAAGCCAATGTGGTCCGC-3′
*Sox9*	Forward: 5′-AACAAGCCACACGTCAAGCG-3′
	Reverse: 5′-GCAGATGCGGGTACTGGTCT-3′
*Col2*	Forward: 5′-GCCCAGATGGCTGGAGGATT-3′
	Reverse: 5′-CCCATGGGACCAGAGACACC-3′
*Aggrecan*	Forward: 5′-ACATCCCAGAAAACTTCTTT-3′
	Reverse: 5′-CGGCTTCGTCAGCAAAGCCA-3′
*PPARγ2*	Forward: 5′-CCTTTACCACGGTTGATTTCTC-3′
	Reverse: 5′-GGCTCTACTTTGATCGCACTTT-3′
*AP2*	Forward: 5′-GCGTAGAAGGGGACTTGGTC-3′
	Reverse:5′-TTCCTGTCATCTGGGGTGATT-3′
*ADIPOQ*	Forward: 5′-TGGTGGATGAGCAGTGGGT-3′
	Reverse: 5′-AGGGTTCAGGACTGGACAGG-3′

### Western blot analysis

Cells were washed with PBS three times and lysed with RIPA containing 1 mM protease inhibitor cocktail and 1 mM phosphatase inhibitor cocktail (Boster). Then 30 μg protein samples were separated by SDS-polyacrylamide gels and transferred to PVDF membranes. The membranes were then blocked with 5% bone serum albumin for 1 h and incubated with appropriate antibodies at 4 °C overnight. Subsequently, blots were incubated with horseradish peroxidase (HRP)-conjugated secondary antibodies for 1 h at room temperature. The bands were detected by the Western ECL Substrate Kit (Thermo Pierce, USA). Protein expressions were determined by mormalizing to GAPDH, and representative bands are shown. Compared to the Control group, the EMF group was treated with EMF for 7 days before culturing in inductive medium.

### Experiment design of tibial nonunion

Seventy-eight 12–13-week-old male SD rats (about 280–320 g) were supplied by the Laboratory Animal Center of Tongji Hospital and were approved by the Committee. Rats were anesthetized by pentobarbital (3.5 mg/100 g weight) administered intraperitoneally. Briefly, an incision was made over the anterior of the right tibia, and muscle was separated by blunt dissection. After conducting a transverse osteotomy of the tibia shaft with an oscillating mini saw, a 21-gauge needle was inserted into the intramedullary tibia shaft from the tibia platform to the distal ankle. Finally, periosteum of 0.5 cm around the fracture was removed, and the nonunion model was finished.

The rats were divided into three groups. For the Control group, the rats were left only tibia nonunions without any treatment. For the Sheet group, osteotomy sites were wrapped with normal cell sheets. For the EMF group, bone fractures were implanted with cell sheets pretreated with SEMF (1 mT, 15 Hz, 4 h/day) for 14 days. At 2, 4, and 6 weeks, six animals from each group were anesthetized, and X-ray photographs were taken to evaluate the bone formation. The X-ray images were scored following the Lane–Sandhu radiographic criteria [26].

### Histological evaluation

At 2, 4, and 6 weeks after X-ray evaluation, six rats from each group were sacrificed. The tibias were harvested and intramedullary pins were removed. Then the specimens were fixed in 4% paraformaldehyde, decalcified, and embedded in paraffin. The tibias were cut longitudinally, stained with hematoxylin and eosin (HE), and prepared for histological evaluation.

### Three-point bending test

At 6 weeks after surgery, six samples from each group were harvested for biomechanical analysis. Tibias from six normal 18-week-old SD rats were introduced as the normal control. A three-point bending test was conducted by an Instron 5566 device (Instron Corporation, Norwood, MA, USA) following the manufacturer’s instructions. Briefly, after extracting the intramedullary pins, the bone was placed horizontally onto the machine with a span distance of 20 mm between the two support points. The pressing force was directed vertically to the bone healing part and applied at 2 mm/min until failure occurred. The ultimate force (*F*), load–time curve, load–displacement curve, and ultimate stress (σ) were obtained.

### Statistical analysis

Values were displayed as the mean ± standard deviation (SD). The proliferation curve of the two groups evaluated at multiple time points was analyzed with two-way analysis of variance (ANOVA) followed by Bonferroni’s multiple comparison test. Other data comparisons were analyzed by one-way ANOVA or Student’s *t* test. Significance was confirmed at *p* < 0.05. All experiments were at least performed three times.

## Results

### Legacy effects of EMF on BMSC proliferation

**Fig. 2:**
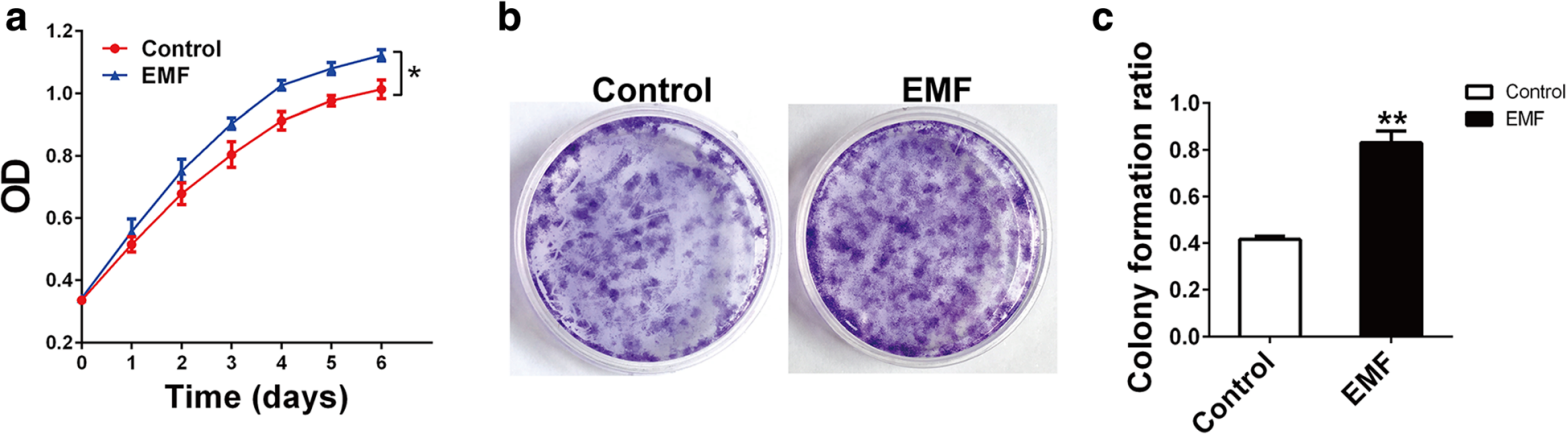
Lasting effects of EMF on BMSC proliferation. **a** BMSCs with or without EMF pretreatment were seeded in 96-well plates, and proliferation of both groups detected from day 0 to day 6 (*n* = 3). **b** Representative gross observation of colony-forming unit formation of both groups after 7 days of culture. **c** Colonies containing more than 50 cells counted, and colony-forming unit ratio acquired by colony number/400 (*n* = 3). Data shown as mean ± SD. *P < 0.05, **P < 0.01. EMF electromagnetic fields, OD optical density

To explore the legacy effects of EMF on BMSC proliferation, we used a CCK-8 assay and conducted a colony-forming assay. In the CCK-8 assay, BMSCs of the EMF group were treated with EMF for 7 days and seeded in 96-well plates for subsequent 6-day culture. In the colony-forming assay, BMSCs of the EMF group were seeded onto a 3.5-cm dish for 7-day culture after 7 days of EMF exposure. For the Control group of the two assays, cells were cultured for the same duration without EMF exposure. Compared with the Control group, BMSCs pretreated with EMF exhibited a higher proliferation level (Fig. 2a). Furthermore, significantly higher formation of colony-forming units was observed in the EMF group (Fig. 2b).We calculated the colony ratio of the two groups, and the EMF group ratio was almost twice as high as that of the Control group (Fig. 2c).

### BMSCs pretreated with EMF showed stronger osteogenic differentiation capacity

**Fig. 3:**
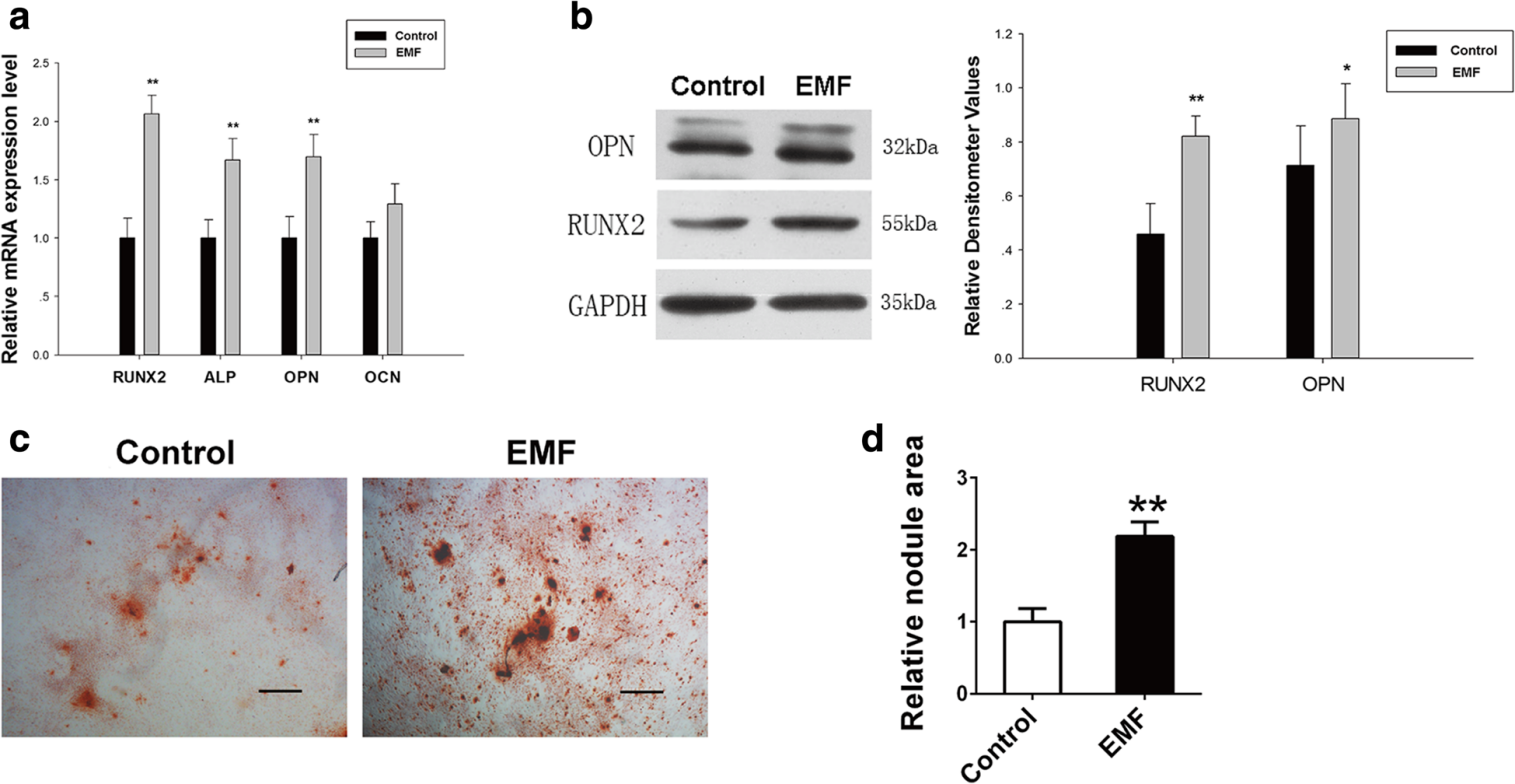
BMSCs pretreated with EMF exhibited stronger osteogenic differentiation potential. **a** RUNX2, ALP, OPN, and OCN mRNA levels of two groups analyzed by RT-PCR. GAPDH used as loading control for quantification (*n* = 3). **b** Expression of OPN and RUNX2 proteins of both groups determined by western blot analysis. Relative densitometer values quantified by ImageJ software, GAPDH used as internal control (*n* = 3). **c** Images of Alizarin Red S staining exhibited plaques of calcified extracellular matrix of both groups. Scale bar = 100 μm. **d** Semi-quantitative analysis of Alizarin Red S staining among both groups (*n* = 6). Data shown as mean ± SD. *P < 0.05, **P < 0.01. EMF electromagnetic field

To determine the lasting effects of EMF on the osteogenic differentiation capacity of BMSCs in vitro, we first examined the expression of osteogenesis-related genes in two groups by RT-PCR. In contrast to the Control group, the EMF group was pretreated with EMF for 7 days before culturing in inductive medium. After 7 days of culture in inductive medium, BMSCs of the EMF group showed an increased expression of RUNX2 (nearly 1-fold), ALP (0.7-fold), and OPN (0.74-fold). No significant difference was seen in the expression level of OCN (Fig. 3a). To further verify the continuous effects of the EMF treatment, western blot analysis was used to measure the osteogenesis-related protein expressions. Compared to the Control group, BMSCs of the EMF group were pretreated with EMF for 7 days before culturing in inductive medium for the subsequent 7 days. Accordingly, the levels of RUNX2 and OPN were boosted after EMF pretreatment (Fig. 3b). We conducted the Alizarin Red S staining, and BMSCs with or without 7 days of EMF exposure were cultured with inductive medium for 14 days. The EMF group exhibited more plaques of calcified extracellular matrix in the microscopic view (Fig. 3c). The corresponding positive staining area of the two groups was analyzed, and same tendency was observed (Fig. 3d).

### The lasting effects of EMF on BMSC chondrogenic differentiation potential

**Fig. 4:**
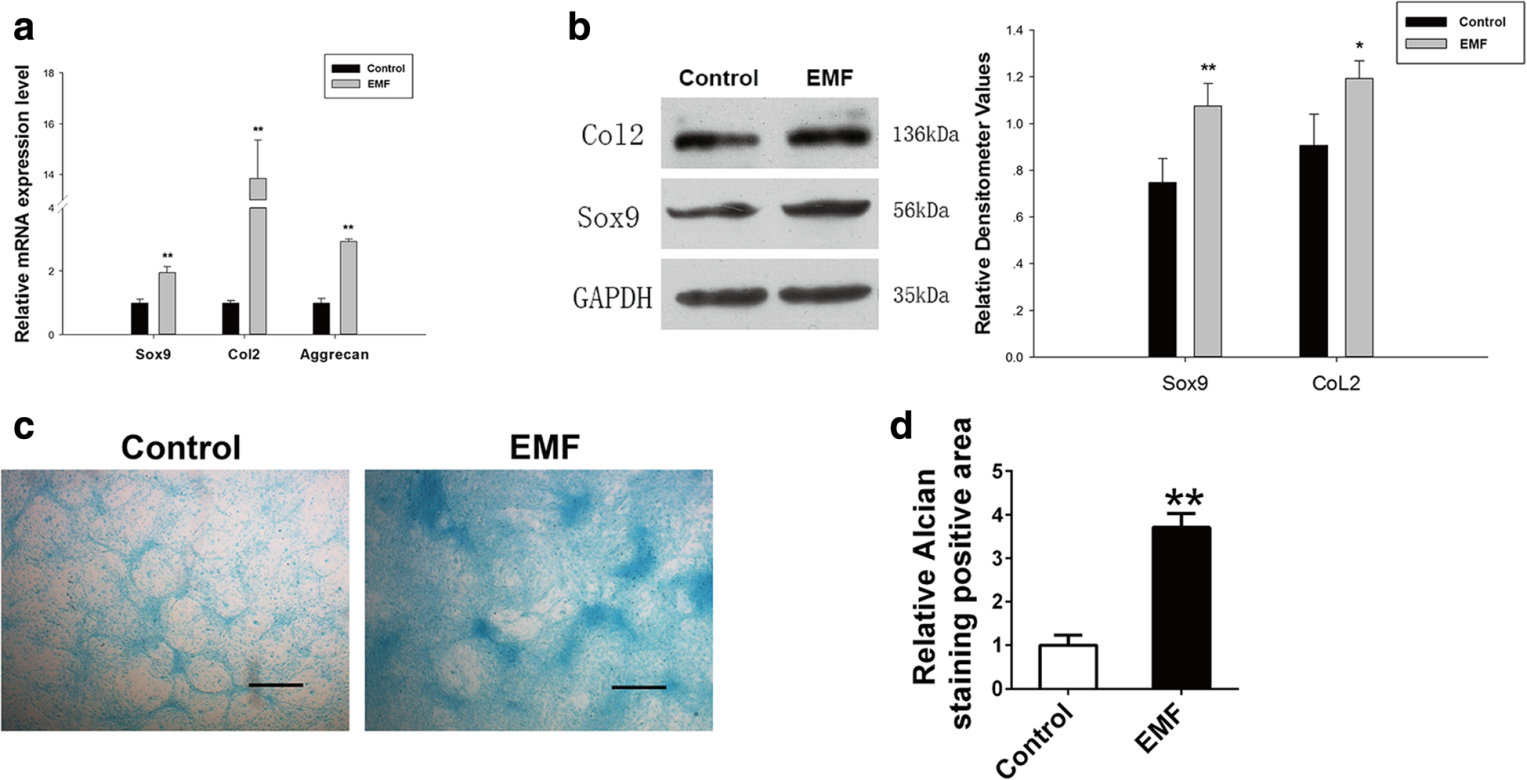
Continuous effects of EMF on BMSC chondrogenic differentiation capacity. **a** SOX9, CoL2, and Aggrecan mRNA levels of two groups determined by RT-PCR. GAPDH used as internal control for quantification (*n* = 3). **b** Expression of Col2 and Sox9 proteins of both groups detected by western blot analysis. Relative densitometer values quantified by ImageJ software, GAPDH served as loading control (*n* = 3). **c** Presentation of Alcian Blue staining of both groups. Scale bar = 100 μm. **d** Semi-quantitative analysis of Alcian Blue staining among both groups (*n* = 6). Data shown as mean ± SD. *P < 0.05, **P < 0.01. EMF electromagnetic field

To confirm the legacy effects of EMF on the chondrogenic differentiation potential of BMSCs, we introduced q-PCR to evaluate the expression of chondrogenesis-related genes of the two groups. Compared with the Control group, BMSCs of the EMF group were pretreated with EMF for 7 days before culturing in inductive medium. After 7 days of culture in inductive medium, BMSCs of the EMF group exhibited an increased expression of Sox9 (almost 1-fold), CoL2 (nearly 14-fold), and Aggrecan (3.2-fold) (Fig. 4a). Furthermore, western blot analysis was conducted to detect the lasting effects of EMF on BMSC chondrogenic differentiation capacity. In contrast to the Control group, the EMF group was pretreated with EMF for 7 days before culturing in inductive medium for the subsequent 7 days. Here, we found that the protein expressions of Sox9 and Col2 were boosted in the EMF group (Fig. 4b). For Alcian Blue staining, BMSCs with or without 7 days of EMF pretreatment were cultured with inductive medium for 14 days. Deeper staining was seen in the EMF group (Fig. 4c). The positive staining area of the two groups was measured, and a similar tendency was observed (Fig. 4d).

### EMF treatment had a continuous impact on BMSC adipogenic differentiation capacity

**Fig. 5:**
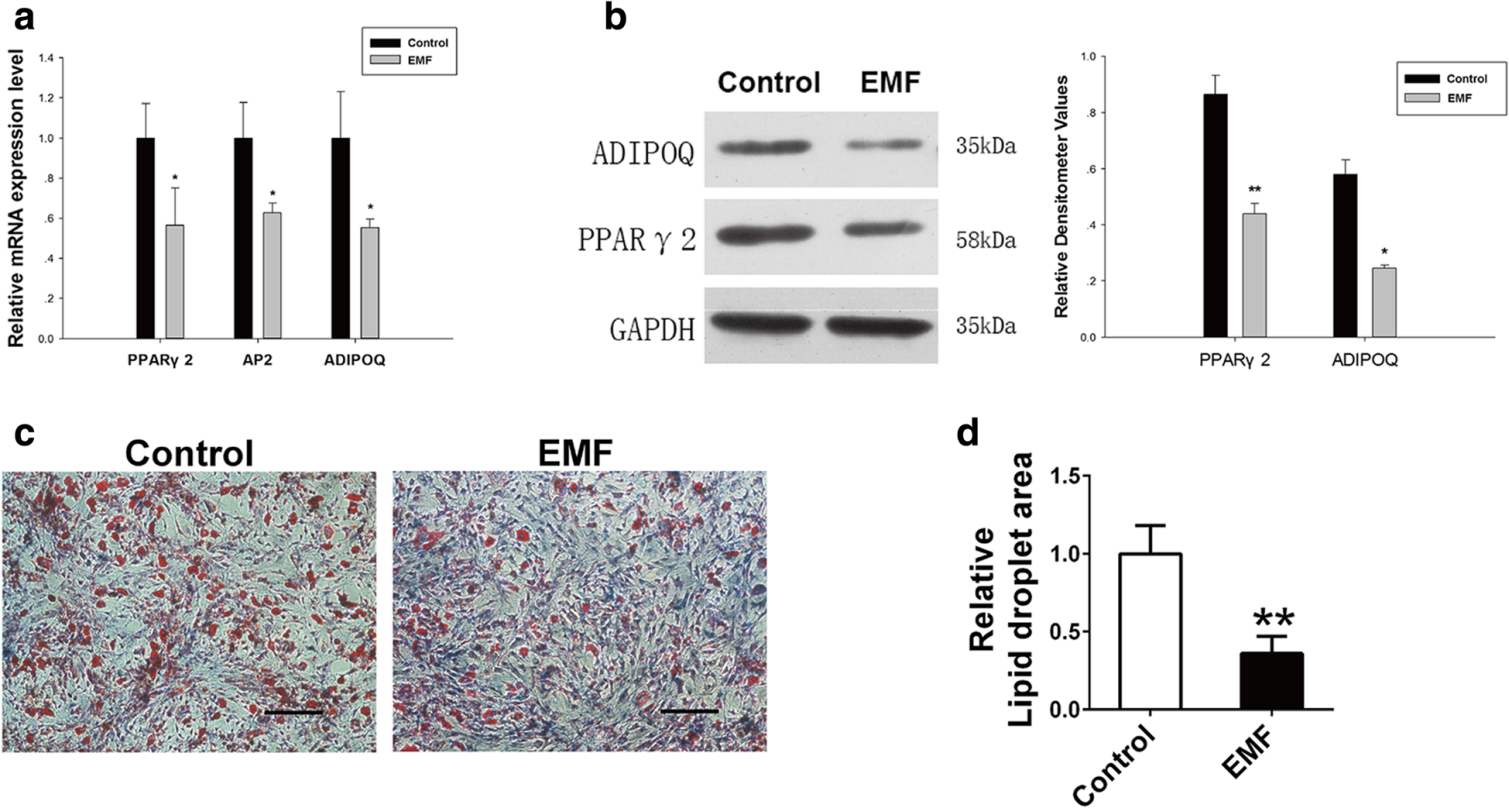
EMF treatment had lasting impact on BMSC adipogenic differentiation potential. **a** PPARγ2, AP2, and ADIPOQ mRNA levels of two groups detected by RT-PCR. GAPDH served as loading control for quantification (*n* = 3). **b** Expression of ADIPOQ and PPARγ2 proteins of both groups determined by western blot analysis. Relative densitometer values quantified by ImageJ software, GAPDH used as internal control (*n* = 3). **c** Images of Oil Red O staining showed lipid droplets of both groups. Scale bar = 25 μm. **d** Semiquantitative analysis of Oil Red O staining among both groups (*n* = 6). Data shown as mean ± SD. *P < 0.05, **P < 0.01. EMF electromagnetic field

To manifest the continuous impact of EMF on BMSC adipogenic differentiation potential, RT-PCR was conducted to detect the expression of adipogenesis-related genes in two groups. Compared to the Control group, the EMF group was pretreated with EMF for 7 days before culturing in inductive medium. After 7 days of culture in inductive medium, BMSCs of the EMF group showed a decreased gene expression of PPARγ2 (0.43-fold), AP2 (0.38-fold), and ADIPOQ (0.41-fold) (Fig. 5a). To further confirm the legacy effects of EMF on BMSC adipogenic differentiation capacity, we introduced western blot analysis to verify the adipogenesis-related proteins. Compared with the Control group, the EMF group was pretreated with EMF for 7 days before culturing in inductive medium for the subsequent 7 days. Accordingly, the expressions of PPARγ2 and ADIPOQ were reduced in the EMF group (Fig. 5b). We performed Oil Red O staining, and BMSCs with or without 7 days of EMF exposure were cultured with inductive medium for 14 days. The EMF group showed fewer lipid droplets (Fig. 5c). The lipid droplet area among the two groups was compared, and same tendency was observed (Fig. 5d).

### EMF exposure had a significant lasting therapeutic effect on rat tibia nonunion combined with BMSC sheets

**Fig. 6:**
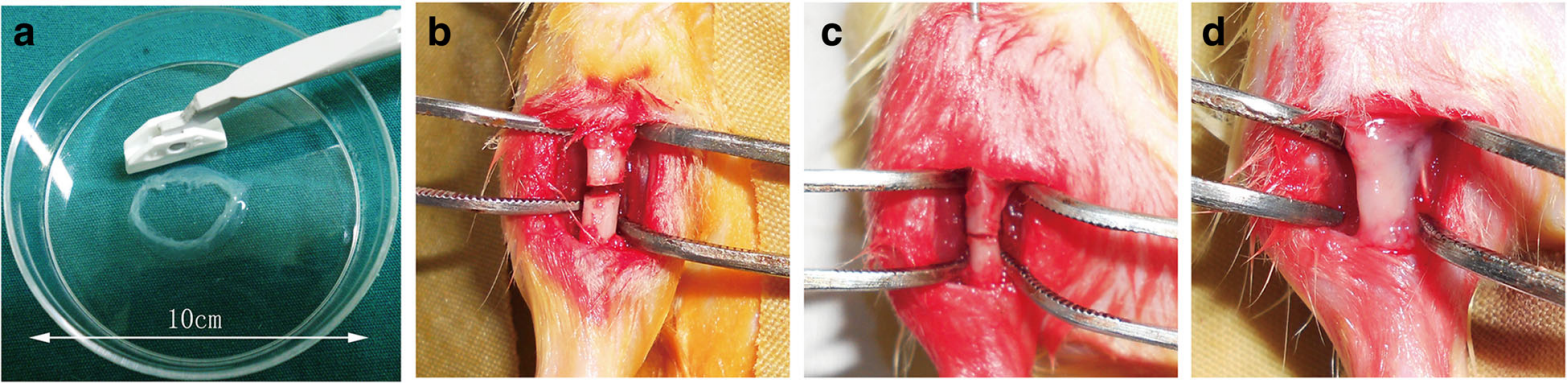
Application of BMSC sheets onto fracture sites in rat tibia nonunion model. **a** Macroscopic image of BMSC sheets detached from 10-cm dishes by scraper. **b** Transverse osteotomy of tibia shaft conducted using oscillating mini saw. **c** Fracture fixed by 21-guage needle and periosteum of 0.5 cm around it removed. **d** Fracture wrapped with BMSC sheets

To evaluate the legacy therapeutic effects of EMF in vivo, we constructed a rat tibia nonunion model combined with using BMSC sheets (Fig. 6). BMSC sheets of the EMF group were pretreated with EMF for 14 days, and the cell sheets of the other two groups were kept in the same conditions without EMF treatment. All rats recovered from the operation and no infections or complications were observed.

**Fig. 7:**
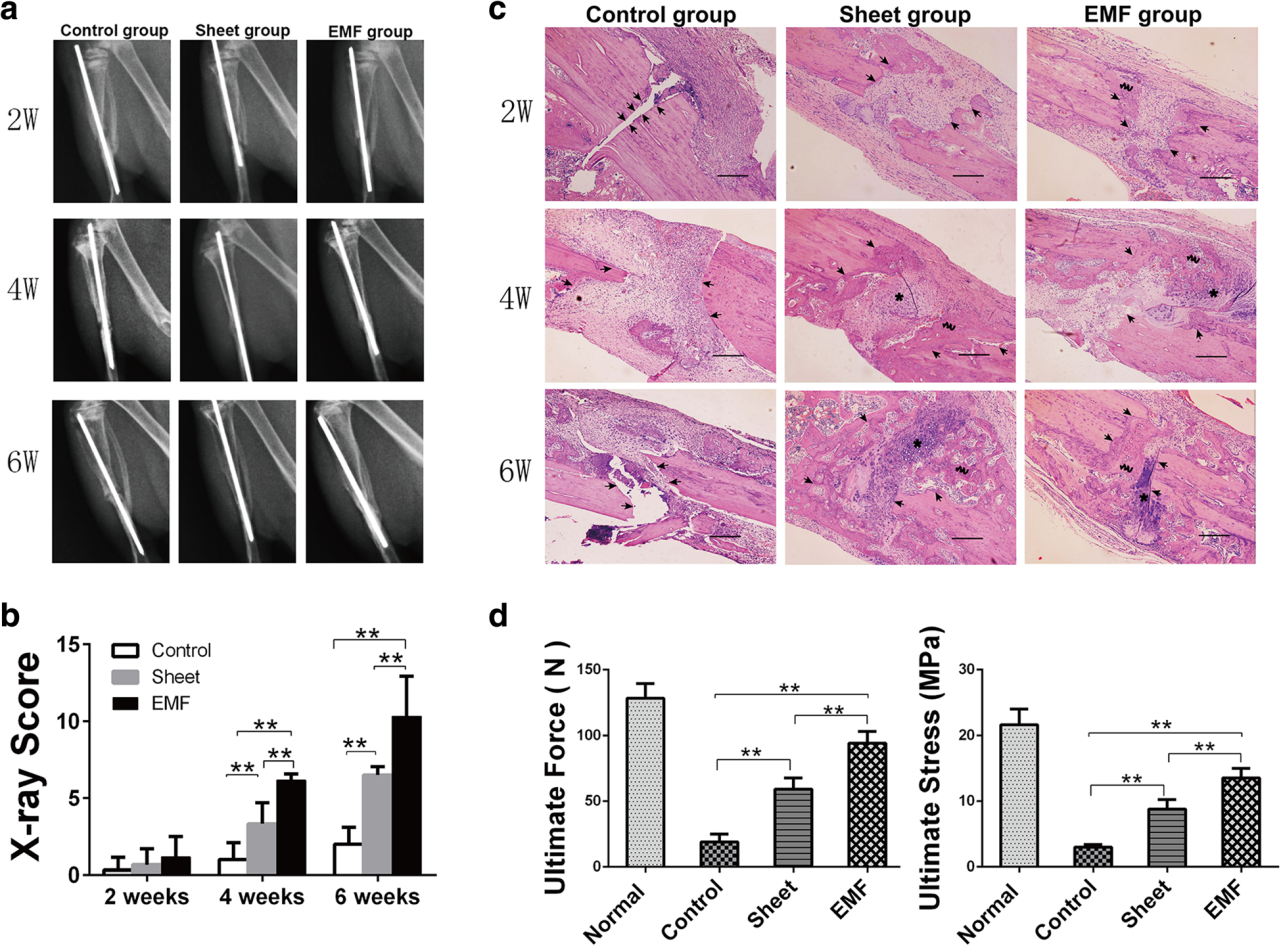
EMF exposure had continuous therapeutic effect on rat tibia nonunion combined with BMSC sheets. **a** Representative X-ray evaluation of tibia fractures in different groups taken 2, 4 and 6 weeks after surgery. **b** X-ray scores of radiographs in different groups at each time (*n* = 6). **c** Longitudinal H&E-stained osteotomy sites in different groups at 2, 4 and 6 weeks after BMSC sheet implantation. Black arrows indicate bone end of the fracture, black wavy lines designate newly formed bone, arrows point to newly formed cartilage. Scale bar = 100 μm. **d** Biomechanical analysis of tibias, comparison of ultimate force and ultimate stress of tibias in different groups at 6 weeks after BMSC sheet implantation (*n* = 6). Data shown as mean ± SD. **P < 0.01. EMF electromagnetic field, W weeks

For gross study of the three groups, we introduced X-ray photographs. Little callus was seen in the Control group after 6 weeks. Fracture bone formation increased time-dependently in the Sheet group and the EMF group. At 6 weeks, the fracture line was not visible in the EMF group (Fig. 7a). Following the Lane–Sandhu radiographic criteria, we scored the X-ray images in each group during different periods. Significantly higher values of radiographic grading scores were observed in the EMF group at 4 weeks and 6 weeks (Fig. 7b).

For histological evaluation, samples of each group at 2, 4, and 6 weeks were stained by HE. Nearly no new bone formation was seen in the Control group after 6 weeks. Newly formed bone increased time-dependently in the Sheet group and the EMF group. In contrast to the Sheet group, more extensive new bone formation was observed especially at 4 and 6 weeks in the EMF group (Fig. 7c).

For biomechanical evaluation, we performed the three-point bending test. At 6 weeks after surgery, results showed that tibias of the EMF group had greater ultimate force and ultimate stress compared to the other two groups (Fig. 7d).

## Discussion

Current strategies for treating bone nonunion faced with unmet achievement [27]. EMF treatment as a noninvasive means has been applied clinically and approved by the US Food and Drug Administration (FDA) [5, 28]. Compared with other treatments, EMF therapy is more convenient and affordable. To uncover the EMF therapy effects, various studies have detected the BMSC proliferation and differentiation capacity combined with the EMF stimulation [6, 29, 30]. However, these research studies mainly focus on the immediate phenomenon after EMF treatment, leaving the legacy effects poorly unraveled. We previously found instantaneous SEMF (15 Hz, 1 mT, 4 h/day) exposure could promote BMSC proliferation and differentiation and we continued to use this parameter [31]. In an in-vitro study, we treated the BMSCs with EMF, and after a period of time the CCK-8 test and colony-forming assay suggested that BMSCs showed enhanced proliferation capacity. For the EMF legacy effects on BMSC multiple differentiation potential, data from RT-PCR, western blot analysis, and corresponding dyeing techniques indicated that BMSCs pretreated with EMF exhibited stronger osteogenic and chondrogenic differentiation potential and weaker adipogenesis capacity.

Although, at present, autologous bone/allograft bone graft with appropriate internal fixation or (and) external fixation is the standard procedure for the treatment of nonunion of fracture [32, 33], many limitations affect the therapeutic effect. These include the limitation of the origin of autogenous bone transplantation, the rejection of allograft, the possibility of spreading disease, and the slow graft absorption. Tissue engineering is a promising approach comprising regulatory factors, seeded cells, appropriate carrier, and adequate blood supply [34, 35]. However, an in-vivo study controlling the morphology, location, and distribution of seeded cells is a challenge [36]. The cell sheet technique, as a scaffold-free graft, guaranteed maximum seeding efficacy and exhibited a unique advantage [15]. Ueyama et al. [37] used osteogenic matrix cell sheets to regenerate maxillofacial defects. Nakamura et al. [17] performed the cell sheet transplantation to treat femur nonunion. Finally, they all achieved remarkable success. Therefore, in our present study, BMSC sheets were selectively applied as the grafts for in-vivo experiment.

To uncover the continuous effects of EMF on BMSCs, in-vitro study is far from enough. It is reported that the BMSC differentiation potential weakened with increasing passage number [38]. Moreover, BMSCs lost stemness during long-term passage [39]. It is easy to assume that BMSCs as grafts would lose efficiency after implantation. Meanwhile, BMSCs should be expanded before application, which restricts their source. We hypothesized that the EMF had a lasting effect on BMSCs. We believe that once the hypothesis is true, deficiency of the source of BMSCs in the clinic and difficulties to regulate the differentiation of grafted BMSCs will be partially handled. Accordingly, we performed the in-vivo experiments. We constructed the EMF pretreated cell sheet and used it as a graft to repair the rat tibial fracture. The X-ray images and radiographic scores indicated that BMSC sheets pretreated with EMF exhibited bigger effects on promoting nonunion healing. Results from the histological evaluation further confirmed the therapeutic effects of EMF pretreatment. In the meantime, the three-point bending test was conducted to evaluate the biomechanical properties of the samples, and tibias of the EMF group showed the greatest toughness. All of these results indicated that the legacy effects of EMF in vivo were significant.

However, EMF with multiple parameters including different durations or various frequencies have diverse effects. We adopted one parameter and more conditions remain to be explored. Furthermore, EMF have been reported to affect the biological process by changing transmembrane ion channels, influencing signal transduction, generating ROS in the cell, and regulating gene expression [22]. The mechanism behind the legacy effects of EMF remains unclear, and further work is needed.

## Conclusions

Besides the immediate effects of EMF on BMSCs, our results suggest that EMF had a lasting impact on BMSCs. Furthermore, a BMSC sheet combined with EMF pretreatment might be a promising way to promote bone nonunion healing, shedding light on clinical strategies. This will give a more complete picture of the EMF biological effects and provide economic benefits for patients with bone disorders.

## References

[1] Borrelli J, Prickett WD, Ricci WM (2003). Treatment of nonunions and osseous defects with bone graft and calcium sulfate. Clin Orthop Relat Res.

[2] Bassett CA, Mitchell SN, Gaston SR (1981). Treatment of ununited tibial diaphyseal fractures with pulsing electromagnetic fields. J Bone Joint Surg Am.

[3] Hannemann PF (2012). The clinical and radiological outcome of pulsed electromagnetic field treatment for acute scaphoid fractures: a randomised double-blind placebo-controlled multicentre trial. J Bone Joint Surg Br.

[4] Tsai MT (2009). Modulation of osteogenesis in human mesenchymal stem cells by specific pulsed electromagnetic field stimulation. J Orthop Res.

[5] Mayer-Wagner S (2011). Effects of low frequency electromagnetic fields on the chondrogenic differentiation of human mesenchymal stem cells. Bioelectromagnetics.

[6] Kaivosoja E (2015). The effect of pulsed electromagnetic fields and dehydroepiandrosterone on viability and osteo-induction of human mesenchymal stem cells. J Tissue Eng Regen Med.

[7] Tabrah FL (1998). Clinical report on long-term bone density after short-term EMF application. Bioelectromagnetics.

[8] Liu HF (2013). Pulsed electromagnetic fields on postmenopausal osteoporosis in Southwest China: a randomized, active-controlled clinical trial. Bioelectromagnetics.

[9] Baek S (2014). Electromagnetic fields mediate efficient cell reprogramming into a pluripotent state. ACS Nano.

[10] Komaki A (2014). Effects of exposure to an extremely low frequency electromagnetic field on hippocampal long-term potentiation in rat. Brain Res.

[11] Tevlin R (2016). Stem and progenitor cells: advancing bone tissue engineering. Drug Deliv Transl Res.

[12] Hutmacher DW (2007). State of the art and future directions of scaffold-based bone engineering from a biomaterials perspective. J Tissue Eng Regen Med.

[13] Zhou Y (2007). Combined marrow stromal cell-sheet techniques and high-strength biodegradable composite scaffolds for engineered functional bone grafts. Biomaterials.

[14] Yorukoglu AC (2017). A concise review on the use of mesenchymal stem cells in cell sheet-based tissue engineering with special emphasis on bone tissue regeneration. Stem Cells Int.

[15] Yang J (2005). Cell sheet engineering: recreating tissues without biodegradable scaffolds. Biomaterials.

[16] Ohki T (2012). Prevention of esophageal stricture after endoscopic submucosal dissection using tissue-engineered cell sheets. Gastroenterology.

[17] Nakamura A (2010). Cell sheet transplantation of cultured mesenchymal stem cells enhances bone formation in a rat nonunion model. Bone.

[18] Shimizu T (2015). The regeneration and augmentation of bone with injectable osteogenic cell sheet in a rat critical fracture healing model. Injury.

[19] Wei F (2012). Vitamin C treatment promotes mesenchymal stem cell sheet formation and tissue regeneration by elevating telomerase activity. J Cell Physiol.

[20] Jiang Z (2017). Laminin-521 promotes rat bone marrow mesenchymal stem cell sheet formation on light-induced cell sheet technology. Biomed Res Int.

[21] Pittenger MF (1999). Multilineage potential of adult human mesenchymal stem cells. Science.

[22] Maziarz A (2016). How electromagnetic fields can influence adult stem cells: positive and negative impacts. Stem Cell Res Ther.

[23] Kim J (2009). Biological characterization of long-term cultured human mesenchymal stem cells. Arch Pharm Res.

[24] Fickert S (2011). Human mesenchymal stem cell proliferation and osteogenic differentiation during long-term ex vivo cultivation is not age dependent. J Bone Miner Metab.

[25] Pountos I (2007). Mesenchymal stem cell tissue engineering: techniques for isolation, expansion and application. Injury.

[26] Lane JM, Sandhu HS (1987). Current approaches to experimental bone grafting. Orthop Clin North Am.

[27] Imam MA (2017). A systematic review of the clinical applications and complications of bone marrow aspirate concentrate in management of bone defects and nonunions. Int Orthop.

[28] Aaron RK, Ciombor DM, Simon BJ (2004). Treatment of nonunions with electric and electromagnetic fields. Clin Orthop Relat Res.

[29] Song M (2014). The effect of electromagnetic fields on the proliferation and the osteogenic or adipogenic differentiation of mesenchymal stem cells modulated by dexamethasone. Bioelectromagnetics.

[30] Ceccarelli G (2013). A comparative analysis of the in vitro effects of pulsed electromagnetic field treatment on osteogenic differentiation of two different mesenchymal cell lineages. Biores Open Access.

[31] Song MY (2014). The time-dependent manner of sinusoidal electromagnetic fields on rat bone marrow mesenchymal stem cells proliferation, differentiation, and mineralization. Cell Biochem Biophys.

[32] Myeroff C, Archdeacon M (2011). Autogenous bone graft: donor sites and techniques. J Bone Joint Surg Am.

[33] Jakoi AM, Iorio JA, Cahill PJ (2015). Autologous bone graft harvesting: a review of grafts and surgical techniques. Musculoskelet Surg.

[34] Bose S, Roy M, Bandyopadhyay A (2012). Recent advances in bone tissue engineering scaffolds. Trends Biotechnol.

[35] Bartold PM (2016). Tissue engineered periodontal products. J Periodontal Res.

[36] Owaki T (2014). Cell sheet engineering for regenerative medicine: current challenges and strategies. Biotechnol J.

[37] Ueyama Y (2016). Maxillofacial bone regeneration with osteogenic matrix cell sheets: an experimental study in rats. Arch Oral Biol.

[38] Bonab MM (2006). Aging of mesenchymal stem cell in vitro. BMC Cell Biol.

[39] Shuai Y (2016). Melatonin treatment improves mesenchymal stem cells therapy by preserving stemness during long-term in vitro expansion. Theranostics.

